# Molecular Subtypes Based on Cell Differentiation Trajectories in Head and Neck Squamous Cell Carcinoma: Differential Prognosis and Immunotherapeutic Responses

**DOI:** 10.3389/fimmu.2021.791621

**Published:** 2021-12-24

**Authors:** Zhen-Dong Huang, Zi-Zhen Liu, Yan-Yi Liu, Yong-Cheng Fu, Lu-Lu Lin, Chao Hu, Hui-Yun Gu, Ren-Xiong Wei

**Affiliations:** ^1^ Department of Spine and Orthopedic Oncology, Zhongnan Hospital of Wuhan University, Wuhan, China; ^2^ Department of Stomatology, Southern Medical University, Guangzhou, China; ^3^ The Third Clinical School, Hubei University of Medicine, Shiyan, China; ^4^ Department of Pathology and Pathophysiology, School of Basic Medicine, Wuhan University, Wuhan, China

**Keywords:** head and neck squamous cell carcinoma, cell differentiation trajectory, single-cell sequencing, molecular subtype, immunotherapy response

## Abstract

**Objective:**

Head and neck squamous cell carcinoma (HNSCC) is one of the most common and lethal malignant tumors. We aimed to investigate the HNSCC cell differentiation trajectories and the corresponding clinical relevance.

**Methods:**

Based on HNSCC cell differentiation-related genes (HDRGs) identified by single-cell sequencing analysis, the molecular subtypes and corresponding immunity, metabolism, and stemness characteristics of 866 HNSCC cases were comprehensively analyzed. Machine-learning strategies were used to develop a HNSCC cell differentiation score (HCDscore) in order to quantify the unique heterogeneity of individual samples. We also assessed the prognostic value and biological characteristics of HCDscore using the multi-omics data.

**Results:**

HNSCCs were stratified into three distinct molecular subtypes based on HDRGs: active stroma (Cluster-A), active metabolism (Cluster-B), and active immune (Cluster-C) types. The three molecular subtypes had different characteristics in terms of biological phenotype, genome and epigenetics, prognosis, immunotherapy and chemotherapy responses. We then demonstrated the correlations between HCDscore and the immune microenvironment, subtypes, carcinogenic biological processes, genetic variation, and prognosis. The low-HCDscore group was characterized by activation of immunity, enhanced response to anti-PD-1/PD-L1 immunotherapy, and better survival compared to the high-HCDscore group. Finally, by integrating the HCDscore with prognostic clinicopathological characteristics, a nomogram with strong predictive performance and high accuracy was constructed.

**Conclusions:**

This study revealed that the cell differentiation trajectories in HNSCC played a nonnegligible role in patient prognosis, biological characteristics, and immune responses. Evaluating cancer cell differentiation will help to develop more effective immunotherapy, metabolic therapy, and chemotherapy strategies.

## Introduction

Head and neck squamous cell carcinomas (HNSCCs) are derived from the mucosal epithelium in the oral cavity, oropharynx, and larynx, and they are mainly associated with tobacco and alcohol consumption (1). HNSCC is the sixth and eighth leading cancer worldwide in terms of incidence rate and mortality rate, respectively, and about two-thirds of HNSCC patients with stage III or IV HNSCC have no evident signs and symptoms (2). There are high recurrence and metastasis rates even after surgical resection due to invasion and metastasis, and the 5-year survival rate of HNSCC patients is only about 40–50%. Chemotherapy, radiation, and combination therapy have been used for the clinical management of HNSCC, but long-term survival rates for most patients with advanced HNSCC remain low. Notably, immunotherapy, such as PD-1 inhibitors and CXCR1/2 inhibitors, has become one of the most promising treatments for HNSCC (3). Studies of HNSCC have shown that the tumor microenvironment (TME) plays an important role in the effects of immunotherapy, as the TME can regulate tumor growth and immune surveillance (4). However, only a minority of HNSCC patients exhibit a positive response to immunotherapy. Multiple factors have been discovered to be involved in the efficacy of PD-1/PD-L1 blockade therapy, such as tumor immunogenicity, T cell function, PD-L1 expression, and intratumor heterogeneity. Thus, more research on molecular subtypes is needed to help accurately determine the heterogeneity subtype of HNSCC patients to identify which patients will respond to immunotherapy (5).

Multiple cells in different developmental states or with distinctly differentiated fates are mixed together when performing bulk RNA-seq, obscuring potential critical molecular events and signals taking place in cell subpopulations. Recent advancements in single-cell RNA sequencing (scRNA-seq) methodologies allow researchers to examine the sequence information from individual cells and have been used to reveal the heterogeneity of cells, dynamic cell differentiation processes, and tumor prognosis (6). Tumor cells exhibit highly heterogeneous, ranging from undifferentiated cells to the cells resembling normal ones. ScRNA-seq method can be used to determine the different states differentiation trajectories of tumor cells to assess how much progress each individual cell has made (7). Recent studies have shown that there is a strong correlation between the cell differentiation trajectories and the heterogeneity of tumor cells in the TME (8). Therefore, the combination of scRNA-seq and bulk-seq technology could help to assess the difference in prognosis between HNSCC patients from the perspective of cell differentiation trajectories. Traditional prognostic indicators include TNM staging and pathological grade, which are mainly based on the clinical pathological characteristics and have limited success in accurately predicting patient prognosis and immunotherapy responses (9). A recent study has shown that molecular subtypes based on TME recognition have provided new insights for customizing immunotherapy regimens for individual cancer patients (10). Thus, it is still necessary to further explore the role of cell differentiation trajectories for predicting immunotherapy responses and survival among HNSCC patients.

In this study, we comprehensively evaluated three distinct molecular subtypes related to cell differentiation trajectories by combining scRNA-seq with bulk RNA-seq, and identified the immune, metabolism, and stemness characteristics among the subtypes. In addition, a prognostic HNSCC cell differentiation score (HCDscore) was developed based on machine-learning models to quantify the differences among individual patients. Our study integrated multi-omics analyses involving genomics, epigenomics, and transcriptomics, which could precisely predict patient prognosis and provide new insights into immunotherapy, metabolic therapy, and chemotherapy.

## Methods and Materials

### Acquisition and Processing of scRNA-Seq Data

The scRNA-seq expression profiling and clinical data of 18 HNSCC cases including 5902 cells were obtained from the GSE103322 dataset in the Gene Expression Omnibus (GEO, https://www.ncbi.nlm.nih.gov/geo/) database. The “Seurat” R package (11) was used to initially process the scRNA-seq expression data. The percentage of mitochondrial genes was calculated by the PercentageFeatureSet function of the “Seurat” R package, and the relationship between sequencing depth and mitochondrial gene sequences was calculated by correlation analysis. Quality control was performed for cells with a gene number <100, sequencing number <50, and mitochondrial gene content >5%. Log transformations were then used to normalize the scRNA-seq expression data, and the top 1500 genes with high variability were selected by the variableFeatures method.

### Dimensionality Reduction and Single-Cell Trajectory Analysis

Significant dimensions with P<0.05 were selected using the principal component analysis (PCA) algorithm, and then the t-distributed stochastic neighbor embedding (t-SNE) algorithm was employed for dimension reduction. The principal components for performing cluster classification analysis across all cells. Differential expression analysis for each cluster with the cutoff criteria of log2[fold change (FC)]>1 and adjusted P-value <0.05 was then performed using the “limma” package (12). The top 10 marker genes with the most significant differences in each cluster were used to create a heatmap. Clusters were determined and annotated using the “SingleR” R package (13) based on the composition patterns of the marker genes.

The functions of “pseudotime” and “trajectory” in the “Monocle” R package (14) were employed to analyze HNSCC cells, with cutoff criteria of log2[fold change (FC)]>1 and adjusted P-value <0.05. Differential expression analysis was performed between branches using the “Monocle” R package, and genes with differential expression levels were designated HDRGs.

### Acquisition and Processing HNSCC Datasets of Bulk RNA-Seq Data

Level 4 gene expression data [Fragments Per Kilobase of transcript per Million mapped reads (FPKM)] from TCGA-HNSC samples were downloaded from the UCSC Xena browser (GDC hub: https://gdc.xenahubs.net). GSE65858 and GSE41613 microarray data on HNSCC samples were downloaded from the GEO database (https://www.ncbi.nlm.nih.gov/geo/). The gene expression data of the TCGA-HNSC cohort were transformed into transcripts per kilobase million (TPM) values, which are more comparable to microarray data. Batch effects due to non-biological experimental factors were reduced using the “ComBat” function in the “sva” R package.

We also obtained clinical data of the TCGA-HNSC, GSE65858, and GSE41613 cohorts, including overall survival (OS), age, gender, smoking status, human papillomavirus (HPV) infection status, TNM stage, cancer stage, and histological type from the UCSC Xena browser and GEO database. Genomic mutation data of the TCGA-HNSC cohort including somatic mutation and copy number variation (CNV) were also obtained from the UCSC Xena database. The “maftools” R package (15) was used to visualize the mutation landscape of the HNSCC cases. For CNV analysis, the Genomic Identification of Significant Targets in Cancer (GISTIC) tool was used to identify significant amplifications and deletions. The CNV gain or loss burden was calculated as the total number of genes with CNV at the focal and arm levels using GenePattern (https://cloud.genepattern.org).

### Unsupervised Clustering of HNSCC Samples Based on HDRGs

Unsupervised clustering analysis was used to determine each patient’s molecular subtype based on HDRGs. The cases were classed based on k-means, with k from 2 to 9, using the “ConsensusClusterPlus” R package (16), with 1000 repetitions to ensure classification stability. The optimal selection of clusters was determined by the consensus matrix and cumulative distribution function (CDF) curve.

### Proportions of Immune Cells Infiltrating in the TME

To quantify the proportions of immune cells in each HNSCC sample, we utilized seven immune cell infiltration estimation algorithms, comprising CIBERSORT (17), MCP-counter (18), EPIC (19), TIMER (20), xCell (21), quanTIseq (22) and IPS (23).

Specifically, CIBERSORT is the most well-recognized method for detecting 22 immune cells based on gene expression by employing linear support vector regression. The microenvironment cell populations (MCP)-counter algorithm evaluated the absolute abundance of eight immune and two stromal cells. The EPIC method detects the fractions of eight immune and cancer cells based on transcriptomic data. The TIMER algorithm provides robust estimation of the infiltration of six immune cells comprising B cells, CD4^+^ T cells, CD8^+^ T cells, neutrophils, macrophages, and dendritic cells in the TME. The xCell algorithm is a gene signature-based method that estimates the abundance scores of 28 immune cell types. The quanTIseq method quantifies the absolute fractions of 10 immune cell types based on bulk RNA-seq data. The IPS algorithm assesses the expression of 28 tumor-infiltrating lymphocytes and subpopulations. In addition, the “ESTIMATE” algorithm (24) was used to comprehensively evaluate the TME components, including the ImmuneScore, StromalScore and tumor purity, for each sample.

### Gene Set Variation Analysis (GSVA)

GSVA was used to quantify activation of signaling pathways by using the “GSVA” R package (25). We obtained biological signatures from the Kyoto Encyclopedia of Genes and Genomes (KEGG) database, the Hallmark gene set v7.1 from the MSigDB database (https://www.gsea-msigdb.org/gsea/msigdb/), 114 metabolism-related gene signatures from a previous study (26), and a typical tumor-related biological process gene set from the “IMvigor210CoreBiologies” R package (27). Finally, GSVA was performed to calculate patient-specific GSVA scores that quantified the pathways or biological processes.

### Gene Function Annotation and Gene Set Enrichment Analysis (GSEA)

Gene function annotation was conducted using the “clusterProfiler” R package (28) with q<0.05 as the cutoff. We also identified gene sets and pathways that were up- and downregulated using GSEA (29). These background gene sets were obtained from the KEGG and MSigDB databases. Upregulated pathways were defined based on enrichment score (ES)>0, while downregulated pathways were defined based on ES<0. Enrichment P values were based on 10,000 permutations and subsequently adjusted using the Benjamini–Hochberg method to control the false discovery rate (FDR).

### Analysis of the TME, Prognosis-Related Metabolic Pathways, and Stemness Indices

The cell infiltration regarding 22 immune cell types inferred from the CIBERSORT algorithm in each HNSCC sample was visualized using the “igraph” R package. Regarding metabolic pathways, GSVA was used to determine the scores for 114 metabolic signatures for each HNSCC sample. To select the prognosis-related metabolic pathways, survival data and univariate Cox regression implemented by the “survival” R package were used, with P<0.05 as the cutoff. We further used the randomSurvivalForest (RSF) algorithm to rank the importance of the prognosis-related metabolic pathways (nrep = 100, which indicates that the number of iterations in the Monte Carlo simulation was 100; nstep = 5). A one-class logistic regression (OCLR) algorithm was used to calculate six stemness imndices (mDNAsi, EREG-mDNAsi, DMPsi, ENHsi, mRNAsi, and EREG-mRNAsi) for each sample (30). We analyzed the differences in stemness indices between the clusters to evaluate the tumor dedifferentiation potential.

### Weighted Gene Co-Expression Network Analysis (WGCNA)

We used the WGCNA algorithm to identify immunity/metabolism/stemness phenotype-related genes by using the “WGCNA” R package (29). HDRGs were selected and then used for the WGCNA. Biweight midcorrelation was used to assess similarity between samples in the WGCNA. A scale-free network was used to select the most suitable β parameter for converting the adjacency matrix into a scale-free topology (soft threshold power β=5, R^2^ = 0.90). A soft-thresholding power of 5 was set for network construction and module detection. In a module–trait analysis, the module eigengene was related, with P<0.05 as the cutoff, to the following three phenotype features: Immunity (ImmuneScore), metabolism (most important metabolic pathways selected from RSF analysis), and tumor stemness (mRNAsi index).

### Analysis of Genomic and Epigenetic Regulation of HNSCC

DNA methylation data from the TCGA-HNSC cohort, obtained using Illumina Infinium Human Methylation 450K BeadChip technology, were downloaded from the UCSC Genome Browser. β-values ranging from 0 to 1 represent the methylation level of each site. Next, a series of quality control algorithms were implemented. Samples with >70% CpG sites missing were excluded. The k-nearest neighbors imputation procedure was used to impute missing data. CpGs located in sex chromosomes and single-nucleotide polymorphisms were removed. We prioritized CpGs in promotor regions (defined as 2 kb upstream to 0.5 kb downstream of transcription start sites). Thereafter, we identified the differential methylation sites (P_adj_<0.05 and |log2FC|>0.15) between different molecular subtypes and visualized them using the “limma” and “pheatmap” R packages.

The DNA damage levels were assessed, including homologous recombination deficiency (HRD), intratumor heterogeneity (ITH), loss of heterozygosity (LOH; number of segments with LOH events, and fraction of bases with LOH events), and aneuploidy score (AS) between different subtypes (31). N6-methyladenosine (m6A) methylation is the most common and abundant RNA epigenetic modifications. A total of 18 m6A regulators comprising 7 writers (METTL3, METTL14, RBM15, RBM15B, WTAP, VIRMA, CBLL1, and ZC3H13), 9 readers (YTHDC1, YTHDC2, YTHDF1, YTHDF2, YTHDF3, IGF2BP1, HNRNPA2B1, HNRNPC, FMR1, LRPPRC, and ELAVL1) and 2 erasers (FTO and ALKBH5) were investigated to observe the level of m6A methylation.

### Construction of HCDscore Based HDRG Signature

We constructed a scoring system to evaluate the impact of individual HNSCC cell differentiation patterns as follows. First, univariate Cox proportional hazards regression was used to identify the significant HDRGs by using the “survival” R package. Genes with P<0.05 were selected as the candidates, which were subjected to LASSO regression to reduce the numbers of predictors. The minimum value of lambda was selected from 1,000 cross-validations in the LASSO regression analysis. A multivariate Cox regression model was established using prognostic HDRGs selected by LASSO-COX regression analysis. The HCDscore was calculated accurately as follows: $\text{HCDscore}=\Sigma_{i=1}^{n}\text{ Coefficient}*\text{Exp},$ where Exp is the expression value of each selected gene.

Next, the optimal cutoff was determined using the “survminer” R package based on the correlation between HCDscore and survival. The samples were then divided into high- and low-HCDscore groups.

### Immunotherapy Response Prediction

First, we compared the expression of 15 immune checkpoint-related genes, which was used to assess the response potentials to immunotherapy in the HNSCC patients. Next, we used the Tumor Immune Dysfunction and Exclusion (TIDE) algorithm (32) to predict responses to immune checkpoint blockade by integrating the expression signatures of T cell dysfunction and T cell exclusion. Besides, an 18 gene “tumor inflammation signature” (TIS) which quantifies an activated immune response in TME, was used to predict response of anti-PD-1 (33). In general, the lower the TIDE score or the higher the TIS score, the better the immunotherapy response. We compared the difference in TIDE and TIS scores between the high- and low-HCDscore groups and the correlation between HCDscore and TIDE, TIS scores.

We also used the subclass mapping (SubMap) method (34) to analyze the similarities of the expression profiles, comparing the identified molecular subtypes with an independent dataset of 47 anti–PD-1 antibody-treated melanoma patients from a longitudinal cohort treated with sequential immune checkpoint blockade (CTLA-4 blockade followed by PD-1 blockade at progression) (35). The lower the P value, the higher the similarity. The “complexHeatmap” R package was used to depict the results.

### Chemotherapy Response Prediction

We predicted the chemotherapy response for each HNSCC sample by training a predictive model on cell line data from the largest publicly available pharmacogenomics database [Genomics of Drug Sensitivity in Cancer (GDSC), https://www.cancerrxgene.org/]. A lower half-maximal inhibitory concentration (IC50), estimated by ridge regression, indicates a better sensitivity to a given drug. The prediction process was performed using the “pRRophetic” R package (36). Specifically, the batch effect was removed using “ComBat”, tissue type was set to “allSoldTumours”, and duplicate gene expression was summarized as the mean value.

### Construction of Nomogram and Verification of Hub Proteins

A nomogram was constructed using the “rms” R package and calibration plots were used to assess the prognostic accuracy of the nomogram. The predicted and actual outcomes of the nomogram were presented in a calibration curve, with the diagonal representing perfect prediction. In addition, the calibration of the prediction model refers to the concordance between the predicted and observed probabilities. Moreover, a GiViTI calibration belt (37) was also constructed to test the goodness of fit of the prediction model. 95% CIs were calculated and displayed in a dark gray area in the plot. More precisely, P>0.05 indicates good model fit. The protein expression of the hub genes in HNSCC and normal paracancerous tissues was verified using immunohistochemical data from the Human Protein Atlas (https://www.proteinatlas.org/).

### Statistical Analysis

The correlations of the TME-infiltrating immune cells were computed using Spearman correlation analyses. One-way analysis of variance (ANOVA) and the Kruskal–Wallis test were used to compare three or more groups. An alluvial diagram was used to visualize the changes in the attributes of individual patients in different clusters. Survival analysis was performed using the “survival” R package. The predictive value of HCDscore for clinical traits and survival was reflected by an ROC curve and the AUC. All P values were two sided and data processing was conducted in R 4.0.1 software.

## Results

### Quality Control and Normalization of scRNA-Seq Data

**Figure 1:**
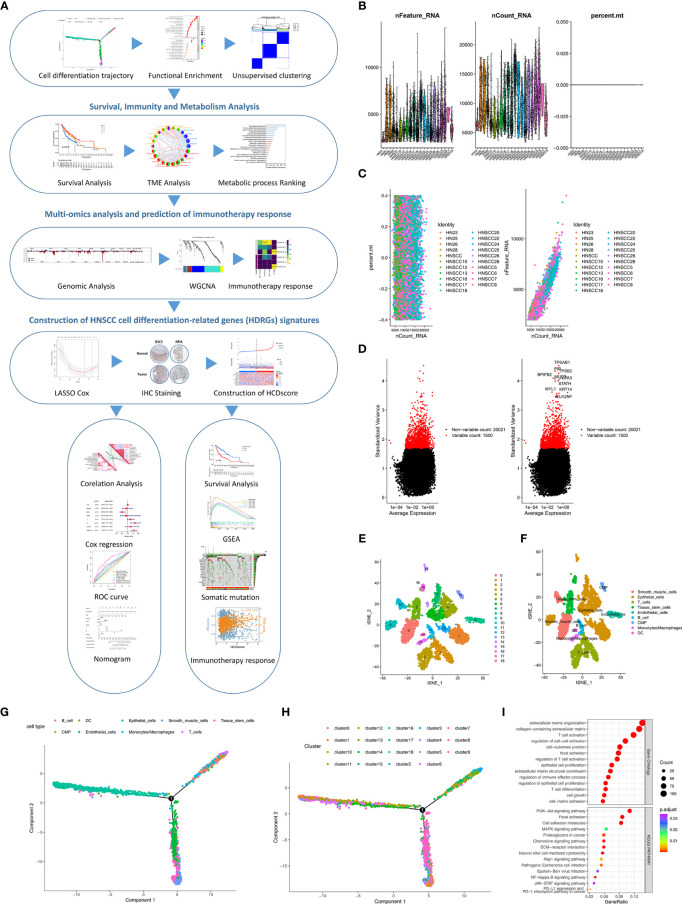
Identification of HDRGs using scRNA-seq analysis. **(A)** Flow diagram of this study’s systematic analysis and validation. **(B)** Quality control including the number of unique genes, the number of total molecules, and the percentage of reads that map to the mitochondrial genome. **(C)** Low correlation of mitochondrial genes across cells and high correlation of gene expression across cells. **(D)** The variance diagram shows 1500 highly variable genes in HNSCC cells. The red dots represent highly variable genes, and the black dots represent non-variable genes. **(E, F)** The t-SNE algorithm was applied for dimensionality reduction with the 19 principal components, and 9 cell clusters were annotated. **(G, H)** Pseudotime and trajectory analysis shows three subsets of HNSCC cells with distinct differentiation patterns. **(I)** GO and KEGG enrichment analysis of HDRGs.

A flow chart was designed to systematically describe the study design (**Figure 1A**). A single-cell RNA-seq dataset from the GSE103322 was subjected to quality control processing and the normalization to exclude nonconforming cells (**Figure 1B**). There was no correlation between mitochondrial gene sequences and sequencing depth (**Figure 1C**). A significant positive correlation between sequencing depth and total intracellular sequences was observed (R=0.93, **Figure 1C**). Among 23,690 genes, 1,500 genes showing high variation were selected for subsequent analysis (**Figure 1D**).

### Identification of HNSCC Cell Trajectory Subsets

PCA method for dimensionality reduction did not lead to clear separations among the HNSCC cells (**Figure S1A**). The top 15 principal components with P<0.05 were selected for further analysis (**Figure S1B**). Next, HNSCC cells were classified into 19 distinct clusters based on the t-SNE algorithm (**Figure 1E**). A total of 5058 marker genes from the 19 clusters were identified by differential analysis, and the top 10% of marker genes in each cluster are shown in a heatmap (**Figure S1C**). These cell clusters were annotated in **Figure 1F**. Subsequently, trajectory analysis was used to project all HNSCC cells onto one root and three branches. The results showed that clusters 6/12/14 were in branch I, mainly containing epithelial cells; clusters 2/10/15 were in branch II, consisting of B cells, monocytes and T cells; and clusters 5/7/8 were in branch III, composed of endothelial cells, smooth muscle cells, and tissue stem cells (**Figures 1G, H**). A total of 811 HNSCC cell differentiation-related genes (HDRGs) were ultimately identified in HNSCC. We further performed functional annotations of the HDRGs from the three distinct cell differentiation branches, and the significantly enriched biological processes are summarized in **Figure 1I**. Enrichment analysis showed that they are involved in immune processes and tumor metastasis-related signaling, such as PD-L1 expression and the PD-1 checkpoint pathway.

### Identification of HDRG-Based Molecular Subtype and Biological Characteristics

**Figure 2:**
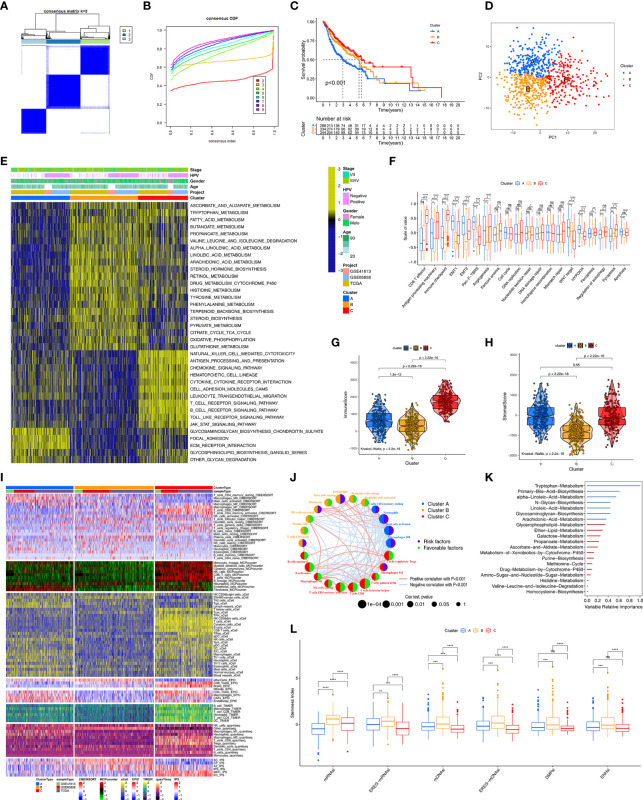
Unsupervised clustering analyses and biological characteristics of each cluster. **(A)** The clustering heatmap corresponding to the consensus matrix for k=3 obtained by consensus clustering. **(B)** CDF plot displays consensus distributions for k=2 to 9. **(C)** Kaplan-Meier curves with Log-rank test shows survival differences for the three clusters including cluster-A, cluster-B and cluster-C based on TCGA-HNSC, GSE41613 and GSE65858 cohorts. **(D)** Principal component analysis for the transcriptome profiles of among three clusters. **(E)** GSVA enrichment analysis shows the activation states of biological pathways among distinct three clusters. The heatmap was used to visualize these biological processes, and yellow represented activated pathways and blue represented inhibited pathways. **(F)** Differences in immune-related and carcinogenic-related signatures among three distinct clusters. The asterisks represented the statistical p value (*P < 0.05; **P < 0.01; ***P < 0.001; ****P < 0.0001; ns, not significant). **(G, H)** Violin plot shows the difference of ImmuneScore and StromalScore from ESTIMATE algorithms in three clusters. **(I)** Heatmap for immune cell and stromal cell infiltration based on CIBERSORT, MCPcounter, xCell, EPIC, TIMER, quanTIseq and iPS algorithms among three clusters. **(J)** The interaction among immune cells. The circle size represented the significance level of P values calculated by Log-rank test. Favorable factors for overall survival are indicated in green, and risk factors indicated in purple. The lines connecting represent immune cells interactions estimated by Spearman correlation analysis, positive correlation is showed in pink and negative correlation in blue. **(K)** 20 prognosis-related metabolic pathways ranked by RSF algorithm. variables with positive variable importance coefficient (blue bars) and variables with zero or negative variable importance coefficient (red bars) are indicated. The absolute value of these importance coefficients (called the variable relative importance) as the horizontal coordinate. **(L)** Grouped boxplot shows the levels of stemness indices in three clusters. The asterisks represented the statistical p value (**P < 0.01; ***P < 0.001; ****P < 0.0001; ns, not significant).

We identified 159 prognosis-related HDRGs using univariate cox regression analysis (**Table S1**). The TCGA-HNSC, GSE65858, and GSE41613 cohorts were employed. The numbers of samples and the clinical baseline and endpoint data of each HNSCC sample are summarized in **Table S2**. Based on the expression of the prognostic-related HDRGs, three distinct molecular subtypes were identified and designated as Cluster-A, Cluster-B, and Cluster-C (k=3, **Figures 2A, B**). **Figure S2A** shows the top10 representative genes in each cluster.

Kaplan–Meier survival analysis demonstrated that Cluster-C exhibited the best survival, whereas Cluster-A had the worst prognosis (p<0.001, **Figure 2C**). In addition, we further verified the effectiveness of unsupervised clustering. First, PCA plot showed that the HNSCC samples were completely distinguished into three clusters (**Figure 2D**). Second, the TCGA-HNSC, GSE65858, and GSE41613 cohorts (as the validation datasets) were employed to perform the clustering analysis with the same algorithm, respectively. As expected, the same trends occurred, indicated the suitability of k=3 (**Figure S2B**). Notably, obvious differences in survival among the three molecular subtypes were observed in the TCGA-HNSC, GSE65858, and GSE41613 cohorts, respectively (**Figure S2C**).

Next, we used the “GSVA” algorithm to explore the biological characteristics of the three distinct molecular subtypes (**Figure 2E**). Cluster-A was markedly enriched in stromal activation pathways such as extracellular matrix receptor interaction and glycosaminoglycan biosynthesis signaling pathways. Cluster-B was enriched in pathways associated with metabolic activation including the activation of tryptophan metabolism, fatty acid metabolism, and drug metabolism involving cytochrome P450. Cluster-C was prominently related to immune activation, including T cell receptor, B-cell receptor, and Toll-like receptor signaling pathways. Subsequently, comparing carcinogenic-related biological processes and immune signatures among the three clusters (**Figure 2F**), we found that Cluster-A, as the stromal activation subtype, was markedly enriched in carcinogenic activation pathways related to epithelial–mesenchymal transition (EMT), transforming growth factor-β (TGF-β), and Wnt-target pathways. CD8^+^ effector T cells, antigen processing machinery, and immune checkpoint were prominently upregulated in Cluster-C, as the immune-activation group. In addition, we found that Cluster-C exhibited the highest levels among the three clusters of the biological processes of ferroptosis and proptosis.

### Immunity, Metabolism, and Stemness Characteristics of the Three Clusters

We further explored the molecular changes, including changes in the TME, metabolic processes, and stemness, underlying the three distinct molecular subtypes (Clusters-A, -B, and -C). We used the ESTIMATE algorithm to calculate the overall fraction of immune cells (ImmuneScore) and stromal cells (StromalScore) in the three molecular subtypes. **Figures 2G** and **S2D** show that Cluster-C exhibited the highest ImmuneScore and lowest tumor purity among the three clusters. **Figure 2H** shows that Clusters-A and -C had a higher StromalScore than Cluster-B, which reflected the characteristic of immune activation in Cluster-C and the abundant stromal components in Cluster-A. To investigate the differences in immune cell infiltration among the three clusters, seven TME cell deconvolution algorithms were used. As shown in **Figure 2I**, Cluster-C had the most abundant anti-tumor immune cell infiltration levels, such as CD8^+^ T cells, macrophages, Th1 cells, NK cells, dendritic cells (DCs), and Th17 cells. Cluster-A had abundant endothelial cells and fibroblast recruitment. **Figure 2J** shows that the TME cell network involved a comprehensive landscape of tumor cell and immune cell interactions and cell lineages, and the Figure shows the effects on the OS of patients with HNSCC.

We also determined the prognostic power of various metabolic pathways. First, 20 prognosis-related metabolic pathways were selected and displayed in **Table S3**. The correlation between those pathways and prognosis was independently analyzed in Cluster-A, Cluster-B, and Cluster-C using the univariate cox regression (**Table S4**). Subsequently, 20 metabolic pathways were ranked by importance, and the tryptophan metabolic pathway was considered to be the most important prognosis-related metabolic pathway in HNSCC (**Figure 2K**). ScRNA-seq technology supports the cancer stem cell theory that posits that cancer stem cells are an important factor in the cause of tumor heterogeneity. Thus, survival analysis was performed and showed that the level of stemness had an important prognostic value in HNSCC (**Figure S2E**). Differences in stemness potential were observed using six stemness indices among the three molecular subtypes (**Figure 2L**). Notably, compared to the other two clusters, Cluster-B had the highest degree of oncogenic dedifferentiation regarding six stemness indices.

### Genomic and Epigenetic Features of Three Clusters Based on HDRGs

**Figure 3:**
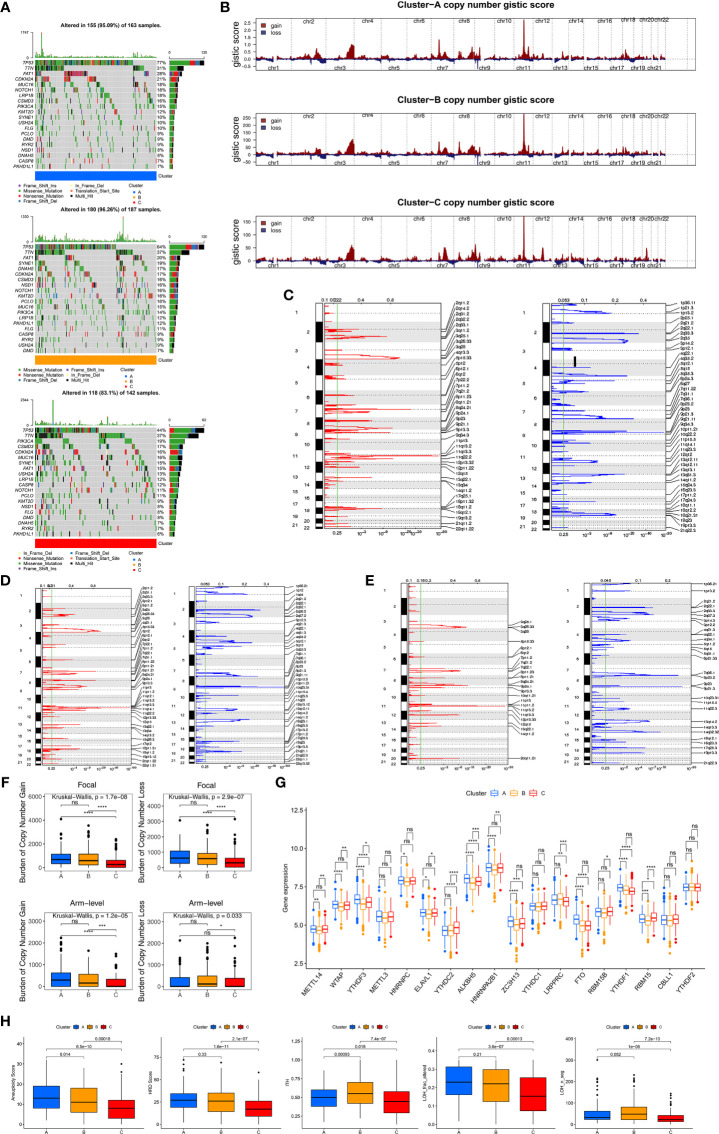
Genomic and epigenetic features and predicting response to immunotherapy. **(A)** The oncoPrint plot shows tumor somatic mutation landscape among three clusters. The upper barplot shows TMB, the number on the right indicated the mutation frequency in each gene. **(B)** Copy number profiles for three clusters, gains were showed in red and losses in blue. Gene segments are placed according to their location on chromosomes, ranging from chromosome 1 to chromosome 22. **(C–E)** Detailed cytoband with focal amplification (left) and focal deletion (right) in the Cluster-A **(C)**, Cluster-B **(D)** and Cluster-C **(E)**, respectively. **(F)** Distribution of and focal and broad (arm-level) copy number alterations in three clusters. **(G)** The difference of expression for m6A regulators among three clusters. Wilcoxon test was used to test statistical difference, *P < 0.05; **P < 0.01; ***P < 0.001; ****P < 0.0001; ns, not significant. **(H)** Difference of genomic scar signatures including aneuploidy, DNA damage including homologous recombination deficiency (HRD), loss of heterozygosity (LOH; number of segments with LOH events, and fraction of bases with LOH events) and intratumor heterogeneity (ITH) were estimated in among clusters.

To further explore the differences in genome abnormalities among the three distinct molecular subtypes based on HDRGs, somatic mutations, copy number alterations, and CNV burden (BCNV) were analyzed. The top 20 mutated genes were plotted in **Figure S3A**. The most significant mutation types were missense mutations, nonsense mutations, and frameshift deletions. In addition, C > T was observed most frequently in single-nucleotide variants. TP53, TTN, and FAT1 were identified as the most commonly mutated genes, with mutation rates of 66%, 35%, and 21%, respectively. We further compared the distributions of somatic mutations among the three molecular subtypes, as shown in **Figure 3A**. Cluster-B had the highest mutation rate (96.26%), followed by Cluster-A (95.09%) and Cluster-C (83.1%). The Tumor Mutation Burden (TMB) quantification analyses showed that Cluster-C was associated with a markedly lower TMB level (**Figure S3B**).

We further observed that the three clusters exhibited CNV amplifications and deletions. **Figure 3B** shows the distribution of the G-score and amplification/deletion frequencies across all chromosomes in the three clusters. Focal amplifications and deletions in various chromosomal regions were detected for Clusters-A (**Figure 3C**), -B (**Figure 3D**), and -C (**Figure 3E**). We further identified significant amplifications at 31 loci and significant deletions at 34 loci in Cluster-A (q<0.05, **Table S5**), significant amplifications at 32 loci and significant deletions at 32 loci in Cluster-B (q<0.05, **Table S6**), and significant amplifications at 19 loci and significant deletions at 24 loci in Cluster-C (q<0.05, **Table S7**). In addition, compared to Clusters-A and -B, Cluster-C had the lowest gain (p<0.05) BCNV both at the arm- and focal-level (**Figure 3F**).

Epigenetic processes, including DNA methylation and various RNA-mediated processes, influence gene expression at the level of transcription. We mainly focused on DNA methylation and m6A methylation, which is one of the most common RNA modifications. We first identified 1630 differential CpG methylation sites among the three clusters and found that Cluster-C had the highest DNA methylation level (**Figure S3C**). We also collected 18 m6A modification regulators to assess the m6A methylation modification level, and we found that Cluster-B had lower levels of the m6A regulators (**Figure 3G**).

As characteristic genomic scar signatures, LOH, HRD, ITH, and AS were analyzed among the three subtypes. We found that LOH, HRD, ITH, and AS were substantially lower in Cluster-C than the others (**Figure 3H**). In summary, the differences in tumor immunogenicity among the three clusters were significant. Our analysis revealed that certain genomic alterations and epigenetics may drive the differences among the three molecular subtypes.

### Responses to Immunotherapy and Chemotherapy Among Three Clusters

**Figure 4:**
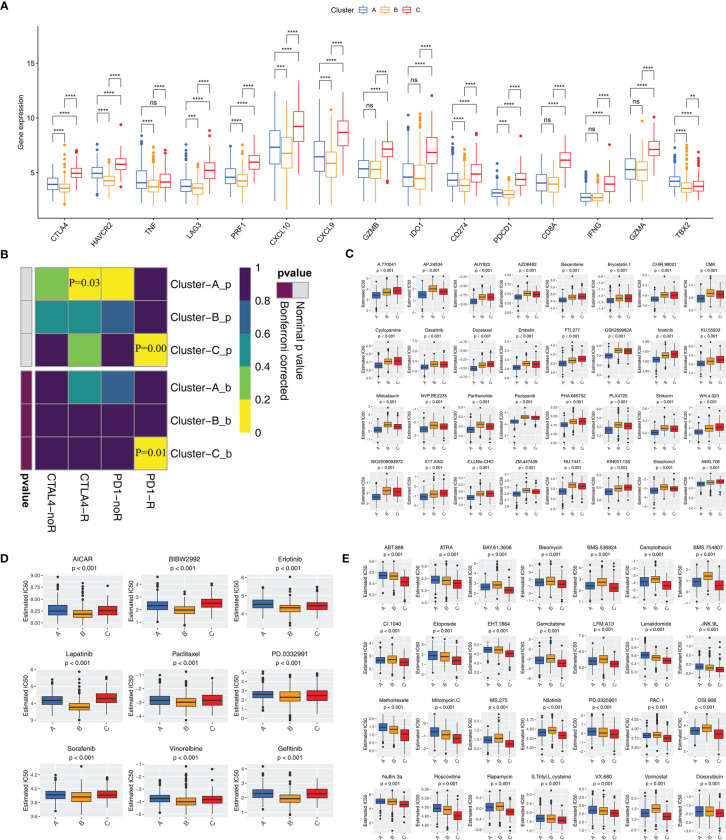
Immune checkpoint inhibitor therapy and chemotherapy responses for each cluster **(A)** Differences in the expression of immune checkpoint genes among three clusters. The statistical difference of clusters was compared using the Wilcoxon test. **P < 0.01; ***P < 0.001; ****P < 0.0001; ns, not significant. **(B)** Submap analysis shows that Cluster-C group could be more respond to anti-PD-1/PD-L1 treatment (Bonferroni corrected P-value = 0.01). **(C–E)** Sensitivity analysis of common chemotherapy drugs in Cluster-A **(C)**, Cluster-B **(D)** and Cluster-C **(E)** groups.

Although blocking immune checkpoints, such as PD-1 and PD-L1, represents a promising approach to treating cancer, some patients are resistant to immunotherapy. We determined which subtype was associated with the largest clinical benefit of immunotherapy. First, we investigated the association between the subtypes and the expression of 15 immune checkpoint-related genes. **Figure 4A** indicates that Cluster-C exhibited higher expression of immune checkpoint genes (except for TBX2) than Clusters-A and -B. Notably, Cluster-C had higher expression of PDCD1, CD274, and CTLA4 than Clusters-A and -B. SubMap algorithm further demonstrated that Cluster-C was more likely to respond to anti-PD-1 antibody treatment (both nominal and Bonferroni-corrected p<0.05) (**Figure 4B**). We assessed the response of the three clusters to 138 chemotherapeutic drugs. Finally, we identified 32 drugs that may be advantageous in Cluster-A (**Figure 4C**), 9 in Cluster-B (**Figure 4D**), and 28 in Cluster-C (**Figure 4E**).

### Identification of Phenotype Related Genes and Clusters

**Figure 5:**
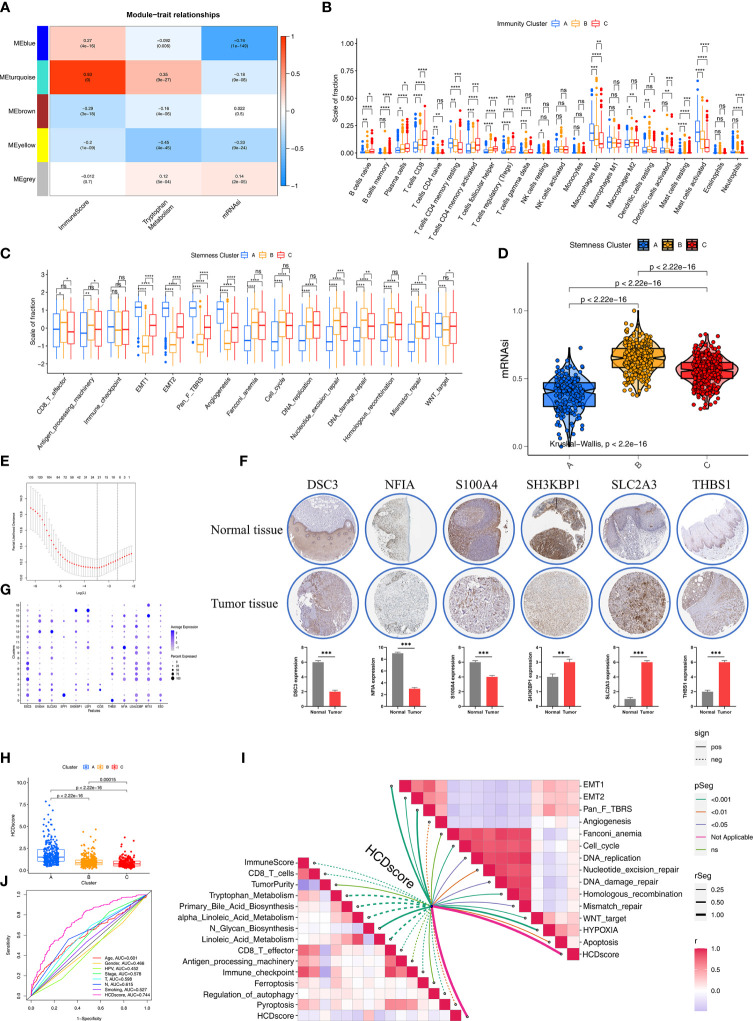
Construction of HDRG signatures and biologic characteristics of HCDscore. **(A)** Correlations between the six gene modules and three clinical traits. Each cell shows the correlation between the gene module and the clinical traits. **(B)** The boxplot shows the difference in the level of 28 immune cells infiltration in the three ImmunityClusters. The asterisks represented the statistical p value (*P < 0.05; **P < 0.01; ***P < 0.001; ****P < 0.0001; ns, not significant). **(C)** The boxplot shows the difference of immune-related and carcinogenic-related signatures in the three ImmunityClusters. **(D)** Violin plot shows differences in mRNAsi stemness indices among three StemnessClusters using Kruskal–Wallis test. The statistical difference of clusters was compared using the Kruskal–Wallis test. **(E)** LASSO Cox regression model construction. λ selection by 10-fold cross-validation. The partial likelihood deviance with changing of log (λ) was plotted. **(F)** Heatmap shows that expression levels of 12 hub genes in 19 clusters. **(G)** The representative protein expression in HNSCC tissue and normal tissue. Data was obtained from the human protein atlas (https://www.proteinatlas.org/). Statistical analysis of these protein expression according to the staining scores of HNSCC and normal tissues. **(H)** Differences in HCDscore among three clusters in HNSCC cohorts. The Kruskal-Wallis test was used to compare the statistical difference. **(I)** Correlations between HCDscore and immune cells, immune-related, metabolic-related, carcinogenic-related or tumor cell death-related signaling pathways. **(J)** The ROC curves comparing the prognostic values of HCDscore and clinical parameters.

To further investigate the specific phenotype-related genes among the HDRGs, WGCNA was performed to identify biologically relevant modules of highly correlated genes. The clustering dendrograms of samples show the module distribution determined by Dynamic Tree Cut and Merged Dynamic (**Figure S4A**). The ImmuneScore (based on the ESTIMATE algorithm), tryptophan metabolic pathway (most important metabolic pathway selected from RSF algorithm), and mRNAsi were selected to define the immunity, metabolism and stemness phenotypes. Five modules were obtained and a heatmap showed the modules associated with these specified phenotypes, that is, MEturquoise for immunity, MEyellow for the metabolic processes, and MEblue for stemness (**Figure 5A**). Ultimately, we identified 310 immune phenotype-related genes, 60 metabolic phenotype-related genes, and 239 stemness phenotype-related genes (**Table S8**). To explore the underlying biological behaviors of phenotype-related subtypes, a consensus clustering algorithm was used based on the immune, metabolic, and stemness phenotype-related genes to further classify the samples into corresponding subtypes. Like the clustering results regarding the three molecular subtypes based on HDRGs, three distinct phenotypes based on the immune, metabolism, and stemness characteristics, designated Immunity A–C, Metabolism A–C, and Stemness A–C, respectively, were identified (**Figure S4B**).

We further explored the different characteristics in the three Immunity, Metabolism, and Stemness subtypes, respectively. In terms of Immunity subtypes, the Immunity-C group had strong infiltration of anti-tumor immune cells, including CD8^+^ T cells, DCs, and Th1 cells (**Figure 5B**) with better survival (**Figure S4C**). In contrast, the Immunity-A group had the opposite trend, with weak infiltration of antitumor immune cells and worse survival (**Figure S4C**). To further investigate the characteristics of the Metabolism A–C subtypes, 114 key metabolism-associated signatures were chosen, based on the results of a previous study (26), and investigated using the GSVA algorithm. **Figure S4D** shows that the Metabolism-B group had significantly higher metabolic processes (especially in terms of amino acid metabolism and fatty acid degradation) than the Metabolism-A and -C groups, and the Metabolism-B group exhibited higher expression related to stromal-related metabolic processes (glycosaminoglycan biosynthesis and hexosamine biosynthesis).

Next, we explored the differences among the three stemness subtypes in terms of biological characteristics. First, we found that the EMT and pan-fibroblast TGF-β response signaling pathways were prominently upregulated in the Stemness A group, while the Stemness B group exhibited strong enrichment of DNA damage repair, DNA replication, and mismatch repair (**Figure 5C**). We also observed differences in mRNAsi, with Stemness B group having the highest level of mRNAsi among the three stemness subtypes (**Figure 5D**). Specifically, the Stemness-A group mainly featured activation of stromal-related processes and the Stemness-B group predominantly featured DNA repair.

### Construction of the HCDscore Based on HDRGs

Given the unique heterogeneity among individuals belonging to the three subtypes, a combination of machine-learning algorithm analysis and Cox proportional hazards regression was used to calculate a score for the cell differentiation pattern of each HNSCC patient, which we designated the HCDscore. To establish the HCDscore, 159 prognosis−related HDRGs were regarded as candidate genes for LASSO regression analysis (**Figure 5E**). Then, 22 HDRGs selected by LASSO regression were used to construct a Cox proportional hazards regression model. Finally, 12 hub genes were identified, and HCDscore were calculated (**Table S9**). Subsequent analysis further explored the performance of the 12 hub genes. In addition, immunohistochemical staining demonstrated the differences in significant hub HDRGs selected by multivariate Cox analysis between HNSCC and normal tissues (**Figure 5F**). **Figure 5G** showed the expression levels of 12 hub HDRGs in 19 clusters identified by scRNA-seq analysis.

**Figure 5H** reveals significant differences in HCDscore among Clusters-A, B, and C. Cluster-A had the highest HCDscore while Cluster-C had the lowest HCDscore, which indicated that low HCDscore was closely linked to immune activation-related processes. To further elucidate the biological significance of HCDscore, we analyzed the correlations of HCDscore with immune, metabolic, and typical biological processes (**Figure 5I**). We found that EMT, pan-fibroblast TGF-β signaling pathways, and DNA damage repair processes were prominently positively correlated with HCDscore, while HCDscore had a strong negative correlation with anti-tumor immune activation, angiogenesis, and immune checkpoint signaling pathways. This suggested that HCDscore may be a risk factor for HNSCC patients.

To quantify the capacity of this scoring system to predict survival, a receiver operating characteristic (ROC) curve was used to observe its predictive accuracy. The HCDscore had a higher area under the curve (AUC) value (0.744) than other clinical parameters (age, gender, HPV infection, TMN stage, cancer staging, smoking status, and histological type), which indicated that the HCDscore had the best predictive ability (**Figure 5J**). We then investigated whether the HCDscore could be used as an independent predictor of HNSCC prognosis by univariate and multivariate Cox regression analyses. As shown in **Figure S4E**, multivariate Cox regression analysis demonstrated that HCDscore was a robust and independent predictor of patients’ prognosis compared to age, gender, HPV infection, TN stage, cancer staging, smoking status, and histological type.

### Identification of the Biological Characteristics of HCDscore

**Figure 6:**
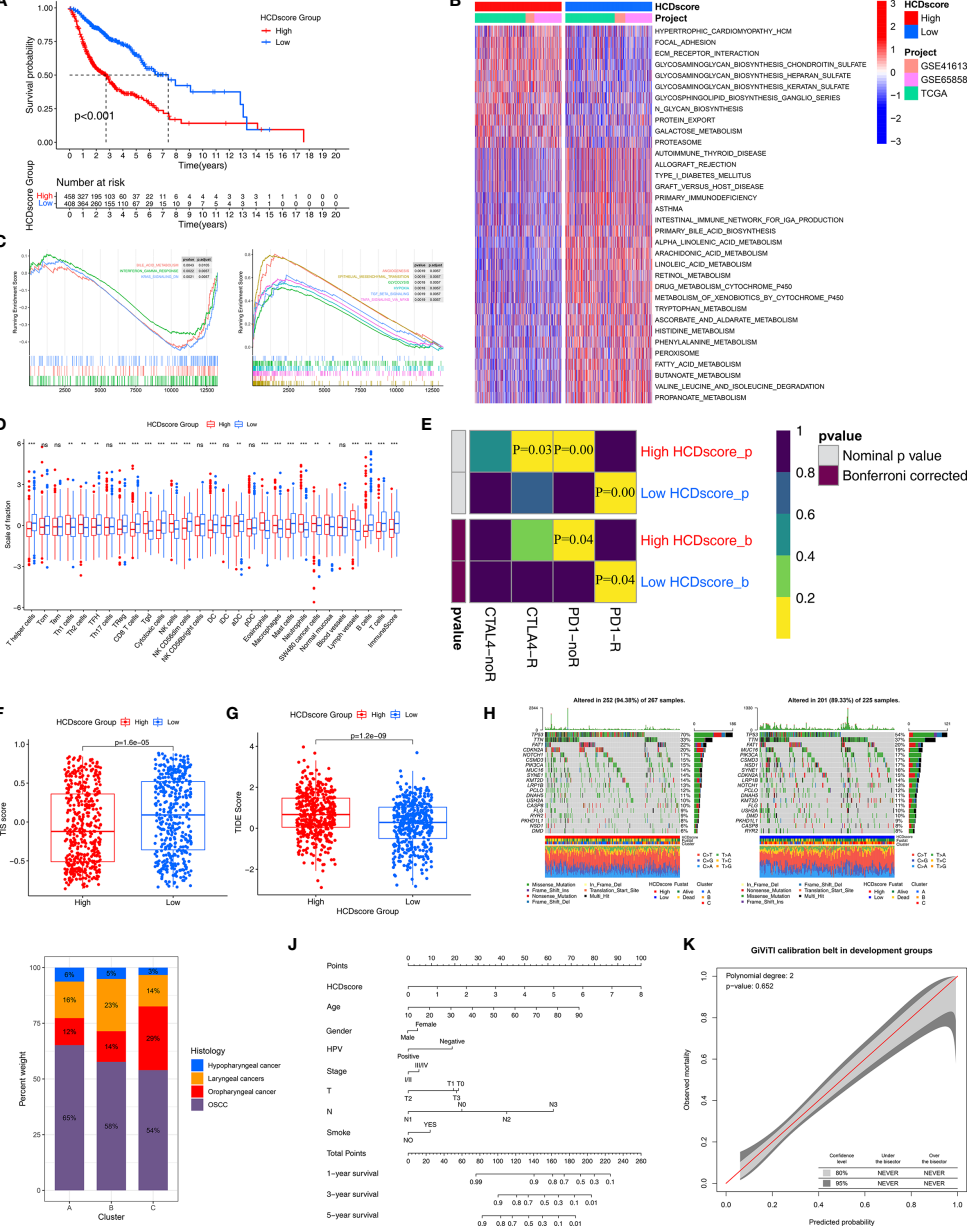
Immunotherapy response prediction and nomogram construction. **(A)** Survival analyses for the low- and high-HCDscore groups using Kaplan–Meier curve and Log-rank test. **(B)** GSVA enrichment analysis shows the activation differences of biological pathways in low- and high-HCDscore groups. The heatmap visualized these biological pathways, and red represented activated pathways and blue represented inhibited pathways. **(C)** GSEA plots show the activated and suppressed gene signatures between the low- and high-HCDscore groups. **(D)** The boxplot shows the difference of the fraction of TME cells and ImmuneScore in low- and high-HCDscore groups. The statistical difference was compared using the Kruskal–Wallis test. *P < 0.05; **P < 0.01; ***P < 0.001; ns, not significant. **(E)** Submap analysis shows that low-HCDscore groups could be more sensitive to anti-PD-1/PD-L1 treatment (Bonferroni corrected P-value = 0.04). **(F, G)** Differences in TIS score and TIDE score between low- and high-HCDscore groups (p < 0.001, Wilcoxon test). **(H)** The oncoPrint plots show tumor somatic mutation in low- and high-HCDscore groups. **(I)** The proportion of HNSCC clinical histopathological type in different clusters. **(J)** A prognostic nomogram predicting 1-, 2-, and 3-year overall survival of HNSCC. **(K)** The calibration curve shows the probability of HNSCC patients in the train group and validation group.

Based on the aforementioned biological processes related to HCDscore, we further determined the clinical outcomes and biological characteristics of patients with different HCDscore levels. First, patients were divided into low- (408 cases) or high- (458 cases) HCDscore groups based on the optimal cutoff value. We found that the low-HCDscore group had better survival (**Figure 6A**). Concurrently, the prognostic value of the HCDscore was validated in the TCGA-HNSC cohort (P<0.001, **Figure S5A**), as well as the GSE65858 and GSE41613 cohorts. The distribution of HCDscore, patterns of survival status and OS, and expression of the 12 hub genes are shown in **Figure S5B**.

Next, to explore the differences in biological behaviors between the high- and low-HCDscore groups, we performed GSVA. As shown in **Figure 6B**, the high-HCDscore group was markedly enriched in stromal and carcinogenic activation pathways such as ECM receptor interaction and glycosaminoglycan biosynthesis. The low-HCDscore group was enriched in pathways associated with metabolism activation. Typical metabolic signatures were further selected to further identify the differences in the metabolic processes between the high- and low-HCDscore groups. The low-HCDscore group was mainly enriched in fat metabolism and amino acid metabolism (**Figure S5C**). GSEA also indicates gene sets associated with tumor promoting effects enriched in the high-HCDscore group (**Figure 6C**), including EMT, TGF-β signaling, angiogenesis, and hypoxia. Subsequent analysis of the TME indicated that the low-HCDscore group had a higher ImmuneScore and was remarkably associated with anti-tumor immune cell infiltration such as CD8^+^ T cells, DCs, and cytotoxic cells (**Figure 6D**).

Given the difference in immune cell infiltration between the low- and high-HCDscore groups, especially regarding CD8^+^ T cells, we further investigated whether HCDscore could predict patients’ responses to immunotherapy. **Figure 6E** shows that the low-HCDscore group was more likely to respond to anti-PD-1 antibody treatment (both nominal and Bonferroni-corrected P=0.04). We found that the low-HCDscore group had a higher TIS score (**Figure 6F**) and a lower TIDE score (**Figure 6G**) than the high-HCDscore group. Correspondingly, HCDscore was significantly positively correlated with TIDE score (P<0.01), and negatively correlated with TIS score (**Figure S5D**).

We next investigated the distributions of somatic alterations in the low- and high-HCDscore groups. By analyzing the mutation annotation files of the TCGA-HNSC cohort, we identified the top 20 mutated genes and displayed them in **Figure 6H**. The mutational landscapes showed that the high-HCDscore group had higher overall somatic mutation rates than the low-HCDscore group. In addition, the most significantly different mutations are listed in a forestplot (**Figure S5E**).

### Correlation Between HCDscore and Histological Subtype, and Nomogram Construction

The histological subtypes of HNSCC in the TCGA-HNSC, GSE65858, and GSE41613 cohorts mainly included oral squamous cell carcinoma (OSCC, 510 cases), oropharyngeal cancer (150 cases), laryngeal cancers (160 cases), and hypopharyngeal cancer (43 cases). The attribute changes of individual patients were displayed in an alluvial diagram in **Figure S5F**. Cluster-C was linked to a low HCDscore and was related to a better outcome. **Figure 6I** showed the distribution of the histological subtypes among Clusters-A, -B, and -C. A stacked column chart also showed the distribution of the histological subtypes in the high- and low-HCDscore groups (**Figure S5G**). Furthermore, **Figure S5H** shows significant difference in HCDscore among OSCC, oropharyngeal cancer, laryngeal cancers, or hypopharyngeal cancer. There were 20 patients with metastatic tumors recorded in all of the TCGA-HNSC, GSE65858, and GSE41613 cohorts, comprising 10 cases in Cluster-A, 4 cases in Cluster-B, and 6 cases in Cluster-C (**Figure S5I**). Kaplan–Meier survival analysis of the four histological subtypes showed no obvious differences (**Figure S5J**). The low-HCDscore group had a better prognosis in each of the individual histological subtypes, except for hypopharyngeal cancer (potentially due to the limited number, 43, of cases) (**Figure S5K**).

Patients with complete clinical data were used to establish a prognostic nomogram predicting 1-, 3-, and 5-year OS based on stepwise Cox regression. HCDscore, age, gender, HPV infection status, tumor stage, TN stage, and smoking status were included in the nomogram (**Figure 6J**). The calibration curves indicated correspondence between the OS predicted by the nomogram and the actual OS of the HNSCC patients (**Figure S5L**). The 95% confidence intervals (CIs) of a GiViTI calibration belt plot did not cross the diagonal bisecting line (P=0.652 in GiViTI calibration test) (**Figure 6K**). Therefore, the predicted probability of the model was consistent with the actual probability, which suggested that the prediction model had strong concordance performance.

## Discussion

HNSCC is an aggressive and heterogeneous neoplasia primarily involving the oral cavity, tonsils, pharynx, and larynx (2). In the past decade, clinical trials of cancer immunotherapy have made remarkable advances in the treatment of a number of malignancies, especially metastatic cancer. Immunotherapy drugs called immune checkpoint inhibitors improved the prognosis in advanced HNSCC patients. Unfortunately, the overall response rate to PD-1 inhibitors for unselected HNSCC patients is only approximately 15–20% due to the intratumor complexity and tumor heterogeneity (38). Although many molecular subtypes of HNSCC have been proposed in recent years, intratumoral and individuals’ heterogeneity are still the greatest challenges in precision cancer therapy. The development of scRNA-seq technologies provides a cell-based resolution method to reveal the transcriptome characteristics of intratumor cells (7). These technologies also provide the statistical power to determine the diverse cellular populations and cell differentiation of tumors. In this study, HNSCC cells with distinct differentiation trajectories were projected into distinct molecular subtypes by combining the results of scRNA-seq and bulk RNA-seq. This study used multi-omics data and clinical data, including gene expression, CNV, somatic mutation, DNA methylation, to explore the characteristics of three molecular subtypes and develop an HDRG scoring system.

To perform HDRG-based molecular typing for HNSCC, we first identified important HNSCC cell differentiation trajectory-related genes using single-cell differentiation trajectory analysis. The Gene Ontology (GO) and KEGG enrichment analyses suggested that the differences in tumor cell differentiation may involve immune- and metabolic-related processes, especially PD-L1 expression and the PD-1 checkpoint pathway. Next, unsupervised clustering analysis based on these genes comprehensively identified three special phenotypes: active stroma, active metabolic, and active immune subtypes, named Cluster-A, -B, and -C, respectively. Notably, Cluster-C had a higher proportion of infiltrating immune cells compared to the other two groups, which mainly related to higher anti-tumor immune cell infiltration, such as CD8+ T cells, DCs, and NK cells, and lower tumor-promoting immune cell infiltration, such as Tregs and gamma delta T (Tgd) cells. The immune cell infiltration network also reflected the denser immune cell interactions in Cluster-C. The immune cell infiltration characteristics of Cluster-C contributed to better survival. In contrast, Cluster-A involved stromal activation accompanied by an immune desert phenotype. We observed that Cluster-A had higher cancer-associated fibroblast (CAF) cell infiltration, endothelial cell infiltration, and activation of pro-tumor biological processes, such as the TGF-β response, EMT, Wnt, and hypoxia pathways. The suppressive activity of T cells promoted the immune escape and progression of tumors in Cluster-A, which also explained the poorer survival in Cluster-A. In accordance with the abundant immune cell infiltration in Cluster-C, we also found that Cluster-C had higher immune checkpoint-related gene expression levels, such as CD274 and PDCD1 levels. The SubMap algorithm also indicated that Cluster-C had better PD-1 inhibitor responses. However, further clinical trials are needed to assess anti-CTLA4 therapy for Cluster-C patients compared to Cluster-A and -B patients. TGF-β signaling has been shown to play an important role in the EMT pathway and is considered as an important step in tumor progression. We thus speculate that the high activation state of EMT and the TGF-β pathway in Cluster-A weakened the response rate to cancer immunotherapy.

Cluster-B is a unique subtype of HNSCC characterized by high levels of metabolic processes, which mainly included amino acid and lipid metabolic processes. These remarkable metabolic characteristics indicate that patients in Cluster-B may benefit from metabolic therapy. We also noticed that Cluster-B involved an immune desert phenotype characterized by high levels of metabolic processes, unlike Cluster-A characterized by stromal activation. In recent years, studies on metabolic reprogramming of HNSCC during immune escape have shown that cancer cells can evolve and develop compensatory metabolic changes to escape death. In light of this, systemic manipulations to direct the tumor cell metabolic status to the normal cell status may reduce the malignancy (39–41). Studies have shown that metabolic therapy for certain metabolic processes provides an alternative for chemotherapy-resistant patients. Studies have also shown that glucose metabolism plays an important role in the occurrence and development of HNSCC. For example, metformin is associated with the prevention of HNSCC (42, 43). Some important metabolic pathways in our study were tryptophan metabolism, primary bile acid biosynthesis, alpha linoleic acid metabolism, and N-glycan biosynthesis. These pathways were correlated with the survival of HNSCC patients (based on the random survival forest ranking) and may provide new insights for future metabolic therapies.

As tumor heterogeneity is focused on in recent years, researchers have been paying increasing attention to the so-called tumor immunological phenotype. According to the spatial distribution of T-cell infiltration in the TME, tumors were divided into different immune profiles including hot tumor and cold tumors (44). Immune-inflamed tumors, also named hot tumors, are mainly characterized by high CD8+ T cells infiltration and expression of PD-1/PD-L1 (45). Immune-excluded tumors and immune-desert tumors can be described as cold tumors. In immune-excluded tumors, CD8+ T cells localize only at invasion margins and do not efficiently infiltrate the tumor. In immune-desert tumors, CD8+ T cells are absent in the tumor. In addition to poor T-cell infiltration, cold tumors are characterized by low PD-1/PD-L1 expression (45). Hot tumors also have a strong infiltration of pre-existing immune cells (e.g. CD8+, DCs, Natural killer immune cells) that facilitate clearance of tumor cells (46). A clinical trial indicated hot tumors have significantly higher expressions of PD-1 and PD-L1 in comparison to cold tumors, they might be more prone to immune checkpoint inhibitors treatments (47). Our results also confirmed that Cluster-C corresponds to abundant CD8+T cell infiltration and highly expressions of PD-1/PD-L1 compared to Cluster-A and Cluster-B. More importantly, Cluster-C characterized by hot tumors has a higher response rate to immune checkpoint inhibitors treatments.

We also analyzed the genomic and epigenetic alterations in the three subtypes. Cluster-C had a lower somatic mutation rate than the other two groups. A pan-cancer study showed that the prognostic value of TMB varies across different cancer types (48), which is consistent with our study. Cluster-C, which had a lower TMB level, had higher survival and immune cell infiltration than the other two groups. A study also revealed that the KL, CCR7, LGR5, and RORB gene expression is associated with low TMB and a favorable prognosis, while immune cell infiltration is related to mutations in these four hub genes (49). As an epigenetic abnormality that can occur in tumors, DNA methylation is considered to be correlated with tumor immune escape signatures (50). In our study, Cluster-C, which had high DNA methylation levels, had high immune cell infiltration levels, which suggests that high DNA methylation levels may promote the infiltration of immune cells in HNSCC. Studies have shown that BCNV can be an important immunogenic activator that promotes the infiltration of immune cells (51, 52). Our results confirmed that patients in the immune activation group (Cluster-C) had a lower BCNV compared to patients in the immune desert groups (Clusters-A and -B) in HNSCC. Altogether, our results showed different immune phenotypes have different genomic characteristics.

To further explore the immunity/metabolism/stemness phenotype genes associated with cell differentiation trajectories, WGCNA was performed. Three unsupervised clustering analyses showed that the immunity phenotype (ImmuneScore)-, metabolism phenotype (tryptophan metabolism)-, and stemness phenotype (mRNAsi)-related genes clustered into three phenotype subtypes, respectively. Each phenotype subtype had unique immunity/metabolism/stemness features, contributing to different prognoses. This suggested the potential influence of tumor cell differentiation trajectories on immunity, metabolism, and tumor stemness. However, the heterogeneity and complexity of individual patients with different HDRG subtypes can easily be ignored; therefore, we constructed an HDRG scoring system designated HCDscore to quantify the differentiation pattern using a series of machine learning algorithms. As expected, HCDscore had many profound clinical implications. First, it was related to tumorigenesis and progression; specifically, it was significantly negatively correlated with anti-tumor immune processes, and positively related to oncogenic signal pathways, such as EMT, Wnt, and hypoxia signaling pathways. Second, there were significant differences in HCDscore between the different molecular subtypes. Third, HCDscore was an independent prognostic factor and exhibited higher prediction accuracy than other clinical parameters in HNSCC. Fourth, HCDscore as a biomarker for predicting immunotherapy response was indicated by analyses involving the TIDE, TIS, and SubMap algorithms. Additionally, HCDscore could also predict drug sensitivity, so it could be used to guide chemotherapy use. Lastly, we combined HCDscore and clinical variables to construct a prognostic nomogram to provide a visual method for predicting OS in HNSCC patients.

However, this study has several limitations. First, although a series of algorithms were used to reduce the potential batch effects as much as possible, the use of the three largest HNSCC databases inevitably led to the neglect of the existence of heterogeneity in the different cohorts. Second, although verified separately in independent cohorts, the results require further large-scale prospective clinical studies to evaluate the effectiveness and practicality of the HCDscore cutoff value. In the current study, the comprehensive evaluation of the cellular, molecular, and genetic factors associated with TME infiltration patterns has yielded several insights that shed light on how tumors respond to immunotherapies and may guide the development of immunotherapy, metabolism, and other drug strategies.

## Data Availability Statement

All data used in this work can be acquired from the Gene-Expression Omnibus (GEO; https://www.ncbi.nlm.nih.gov/geo/ under the accession numbers GSE103322, GSE41613 and GSE65858) and the GDC portal (https://portal.gdc.cancer.gov/).

## Author Contributions

H-YG, CH, and R-XW conceived and designed this study. Z-DH, Z-ZL and Y-CF carried out the analysis procedure. Z-DH, Y-YL and H-YG analyzed the results. Z-DH, Y-YL, L-LL and CH contributed analysis tools. Z-DH, Z-ZL and R-XW participated in the manuscript writing. All authors contributed to the article and approved the submitted version.

## Conflict of Interest

The authors declare that the research was conducted in the absence of any commercial or financial relationships that could be construed as a potential conflict of interest.

## Publisher’s Note

All claims expressed in this article are solely those of the authors and do not necessarily represent those of their affiliated organizations, or those of the publisher, the editors and the reviewers. Any product that may be evaluated in this article, or claim that may be made by its manufacturer, is not guaranteed or endorsed by the publisher.

## Supplementary Material

Supplementary Figure S1 Dimensionality reduction analysis for scRNA-seq data **(A)** PCA analysis based on scRNA-seq data in HNSCC samples. **(B)** PCA identified the 15 PCs with an estimated P value < 0.05. **(C)** The top marker genes from identified differential analysis of each cell cluster are displayed in the heatmap. The colors from purple to yellow indicate the gene expression levels from low to high.

Supplementary Figure S2 Validation of molecular subtypes in multiple cohorts. **(A)** Heatmap shows a representative top10 genes for Cluster-A, Cluster-B and Cluster-C. **(B)** Consensus matrix and CDF plots using unsupervised clustering in TCGA-HNSC (left), GSE65858 (middle) and GSE41613 (right) cohorts, respectively. **(C)** Survival analyses for the three clusters in TCGA-HNSC (left), GSE65858 (middle) and GSE41613 (right) cohorts, respectively. **(D)** Violin plot shows the difference of tumor purity in three clusters. **(E)** Kaplan–Meier curves for each type of six stemness indices using the Log-rank test. The high or low level of stemness indices was defined by optimal cut-off using “survminer” R package.

Supplementary Figure S3 Genomic and epigenetic features. **(A)** The summary of the overall distribution of mutation in HNSCC including the classification of variants, the types of the variants, the type of nucleotide change, the distribution of the variants and top 20 genes with the highest counts of the variants. **(B)** Boxplot shows the difference of levels of tumor mutation burden in the three clusters using the Kruskal–Wallis test. **(C)** CpG sites are displayed for cluster-A, -B and -C. Hypo- and hyper-methylation CpG sites are represented by red and blue bars.

Supplementary Figure S4 WGCNA and identifying phenotype-related subtypes. **(A)** Hierarchical clustering dendrograms of identified co-expressed genes in modules. The branches of the cluster dendrogram correspond to the different gene modules. Five merged modules were identified. **(B)** The consensus matrix heatmaps for k=3 obtained by consensus clustering. **(C)** Kaplan–Meier curves using the Log-rank test for Immunitycluster-A, -B and -C. **(D)** The difference of metabolic pathways among Metabolismcluster-A, -B, -C groups. **(E)** Forest plot using multivariate cox regression analysis of HCDscore and different clinical feature in HNSCC.

Supplementary Figure S5 Histological type analysis and verification of the accuracy of the nomogram. **(A)** Survival analyses for the low- and high-HCDscore groups in TCGA-HNSC, GSE65858 and GSE41613 cohorts, respectively. The optimal cutpoint for the levels of HCDscore was determined by R package “survminer”. **(B)** Distribution of HCDscore, different clusters of survival status and survival time between the high- and low-HCDscore groups. Heatmap shows the expression levels of the 12 hub genes for HNSCC patients. **(C)** GSVA enrichment analysis shows the activation states of metabolism-related biological pathways in high- and low-HCDscore groups. **(D)** The correlation between TIDEscore or TISscore and HCDscore by Spearman correlation analysis. **(E)** Forest plot of the differentially mutated genes between high- and low-HCDscore groups. **(F)** Alluvial diagram shows the changes of cluster, histology type, HCDscore level and survival outcomes. **(G)** The proportion of HNSCC patients with different clinical histopathological type. **(H)** Boxplot shows differences in HCDscore among different clinical histopathological type in HNSCC patients. The statistical difference of clusters was compared using the Kruskal–Wallis test. **(I)** Pie chart of the HNSCC patients with metastasis distribution among three clusters. **(J)** Survival analyses for HNSCC patients with each clinical histopathological phenotype using Kaplan–Meier curve and Log-rank test. **(K)** Survival analyses for each histology type including in HNSCC patients using Kaplan–Meier curve and Log-rank test. **(L)** The calibration plot for internal validation of the nomogram.

Supplementary Table S1 Prognostic analysis of 159 HNSCC cell differentiation-related genes using a univariate Cox regression model.

Supplementary Table S2 The clinical baseline and endpoint data of each HNSCC sample in TCGA-HNSC, GSE41613 and GSE65858 cohorts.

Supplementary Table S3 20 prognosis-related metabolic pathways selected by the randomSurvivalForest algorithm.

Supplementary Table S4 20 metabolic pathways and prognosis was independently analyzed in Cluster-A, Cluster-B, and Cluster-C using the univariate cox regression.

Supplementary Table S5 Amplification or deletion genes for copy number profiles in cluster-A group.

Supplementary Table S6 Amplification or deletion genes for copy number profiles in cluster-B group.

Supplementary Table S7 Amplification or deletion genes for copy number profiles in cluster-C group.

Supplementary Table S8 Immune phenotype-related genes, metabolism phenotype-related genes, and stemness phenotype-related genes selected by WGCNA.

Supplementary Table S9 Identification of hub cell differentiation-related genes using multivariate Cox regression.
