# Supplementary figures and images for: Molecular Subtypes Based on Cell Differentiation Trajectories in Head and Neck Squamous Cell Carcinoma: Differential Prognosis and Immunotherapeutic Responses
Source: Front Immunol. 2021 Dec 24;12:791621. doi: 10.3389/fimmu.2021.791621 (PMC8739483; doi:10.3389/fimmu.2021.791621)

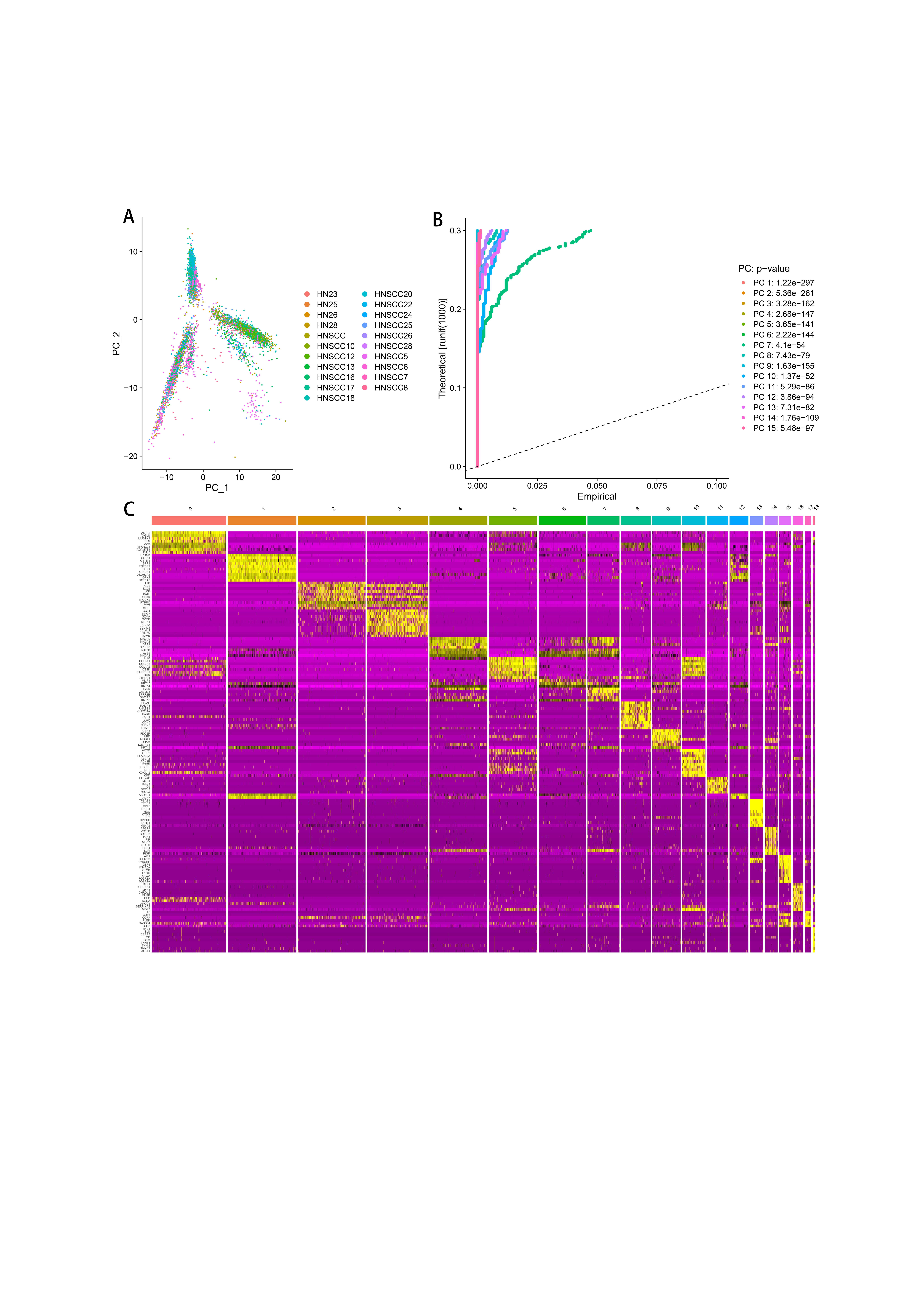

Supplement: Supplementary Figure S1 — Dimensionality reduction analysis for scRNA-seq data (A) PCA analysis based on scRNA-seq data in HNSCC samples. (B) PCA identified the 15 PCs with an estimated P value < 0.05. (C) The top marker genes from identified differential analysis of each cell cluster are displayed in the heatmap. The colors from purple to yellow indicate the gene expression levels from low to high. [file Image_1.tif]

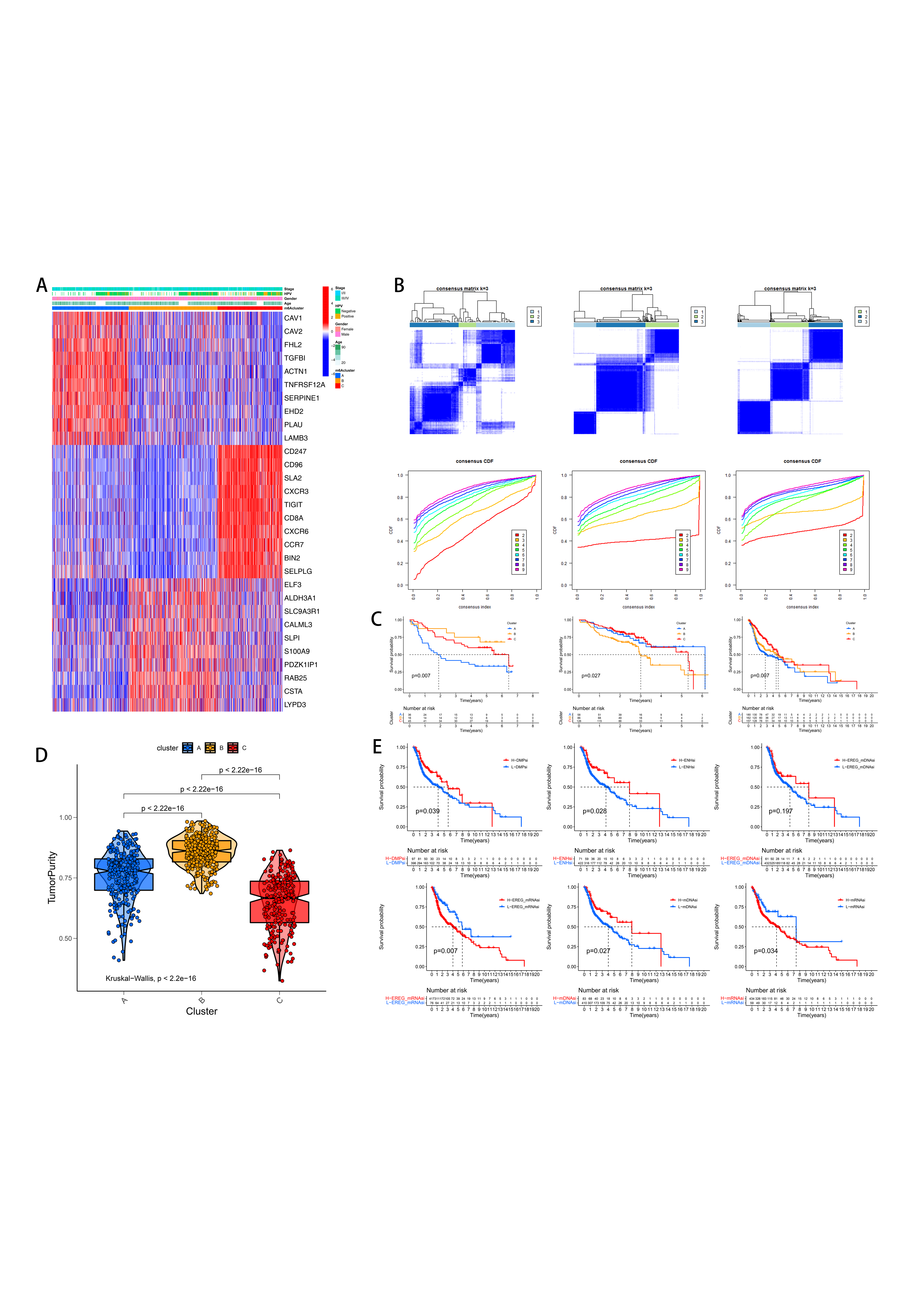

Supplement: Supplementary Figure S2 — Validation of molecular subtypes in multiple cohorts. (A) Heatmap shows a representative top10 genes for Cluster-A, Cluster-B and Cluster-C. (B) Consensus matrix and CDF plots using unsupervised clustering in TCGA-HNSC (left), GSE65858 (middle) and GSE41613 (right) cohorts, respectively. (C) Survival analyses for the three clusters in TCGA-HNSC (left), GSE65858 (middle) and GSE41613 (right) cohorts, respectively. (D) Violin plot shows the difference of tumor purity in three clusters. (E) Kaplan–Meier curves for each type of six stemness indices using the Log-rank test. The high or low level of stemness indices was defined by optimal cut-off using “survminer” R package. [file Image_2.tif]

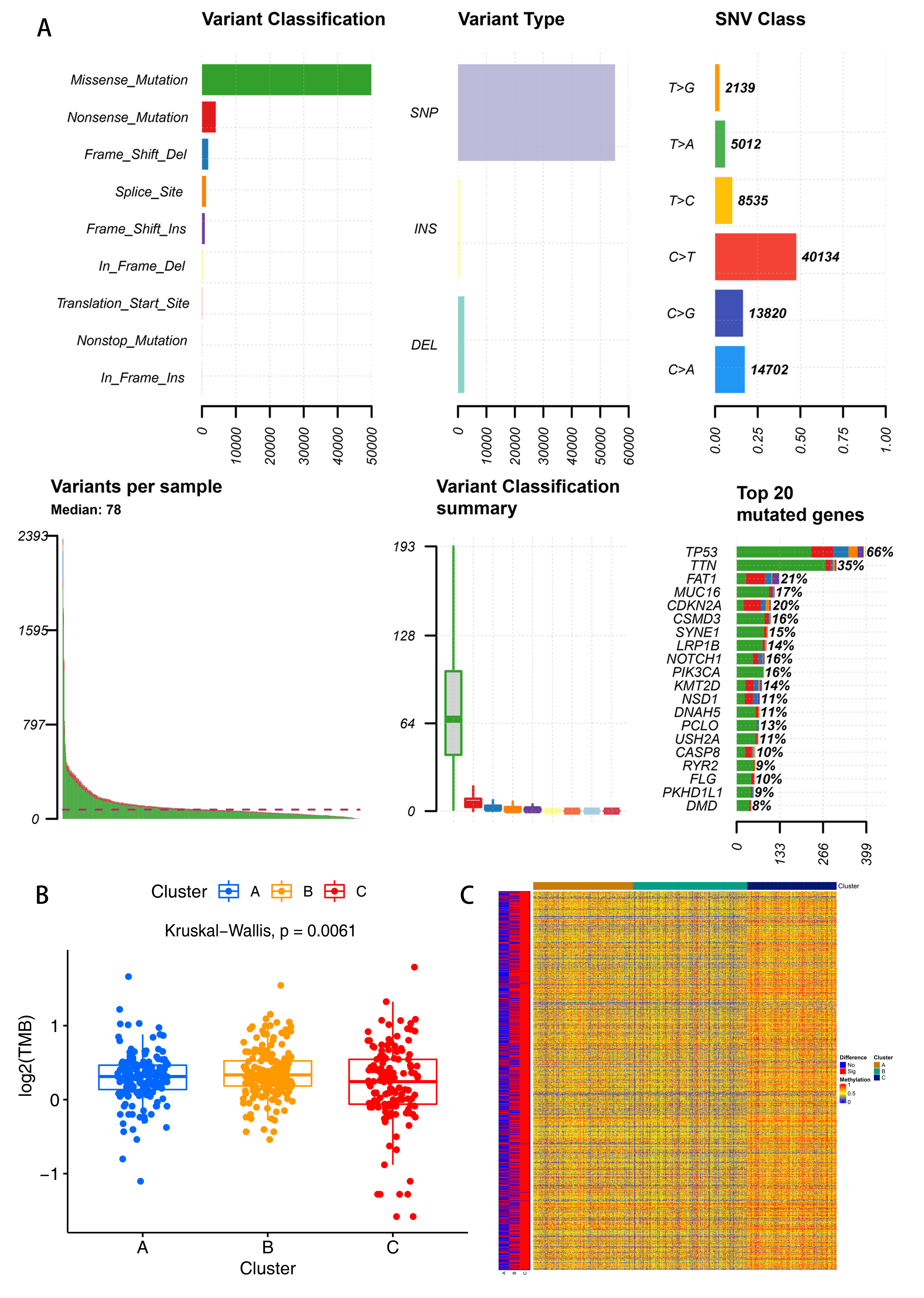

Supplement: Supplementary Figure S3 — Genomic and epigenetic features. (A) The summary of the overall distribution of mutation in HNSCC including the classification of variants, the types of the variants, the type of nucleotide change, the distribution of the variants and top 20 genes with the highest counts of the variants. (B) Boxplot shows the difference of levels of tumor mutation burden in the three clusters using the Kruskal–Wallis test. (C) CpG sites are displayed for cluster-A, -B and -C. Hypo- and hyper-methylation CpG sites are represented by red and blue bars. [file Image_3.tif]

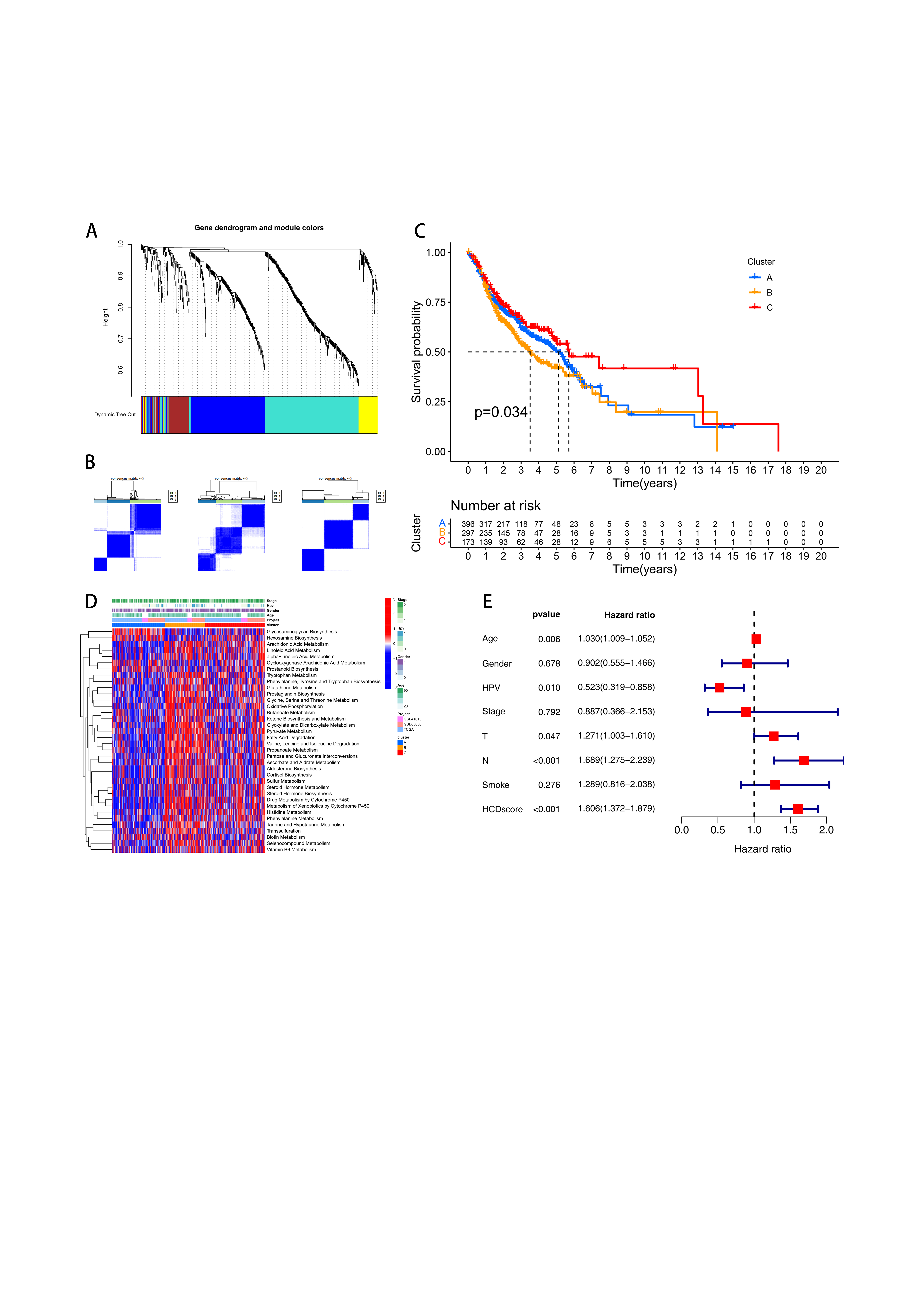

Supplement: Supplementary Figure S4 — WGCNA and identifying phenotype-related subtypes. (A) Hierarchical clustering dendrograms of identified co-expressed genes in modules. The branches of the cluster dendrogram correspond to the different gene modules. Five merged modules were identified. (B) The consensus matrix heatmaps for k=3 obtained by consensus clustering. (C) Kaplan–Meier curves using the Log-rank test for Immunitycluster-A, -B and -C. (D) The difference of metabolic pathways among Metabolismcluster-A, -B, -C groups. (E) Forest plot using multivariate cox regression analysis of HCDscore and different clinical feature in HNSCC. [file Image_4.tif]

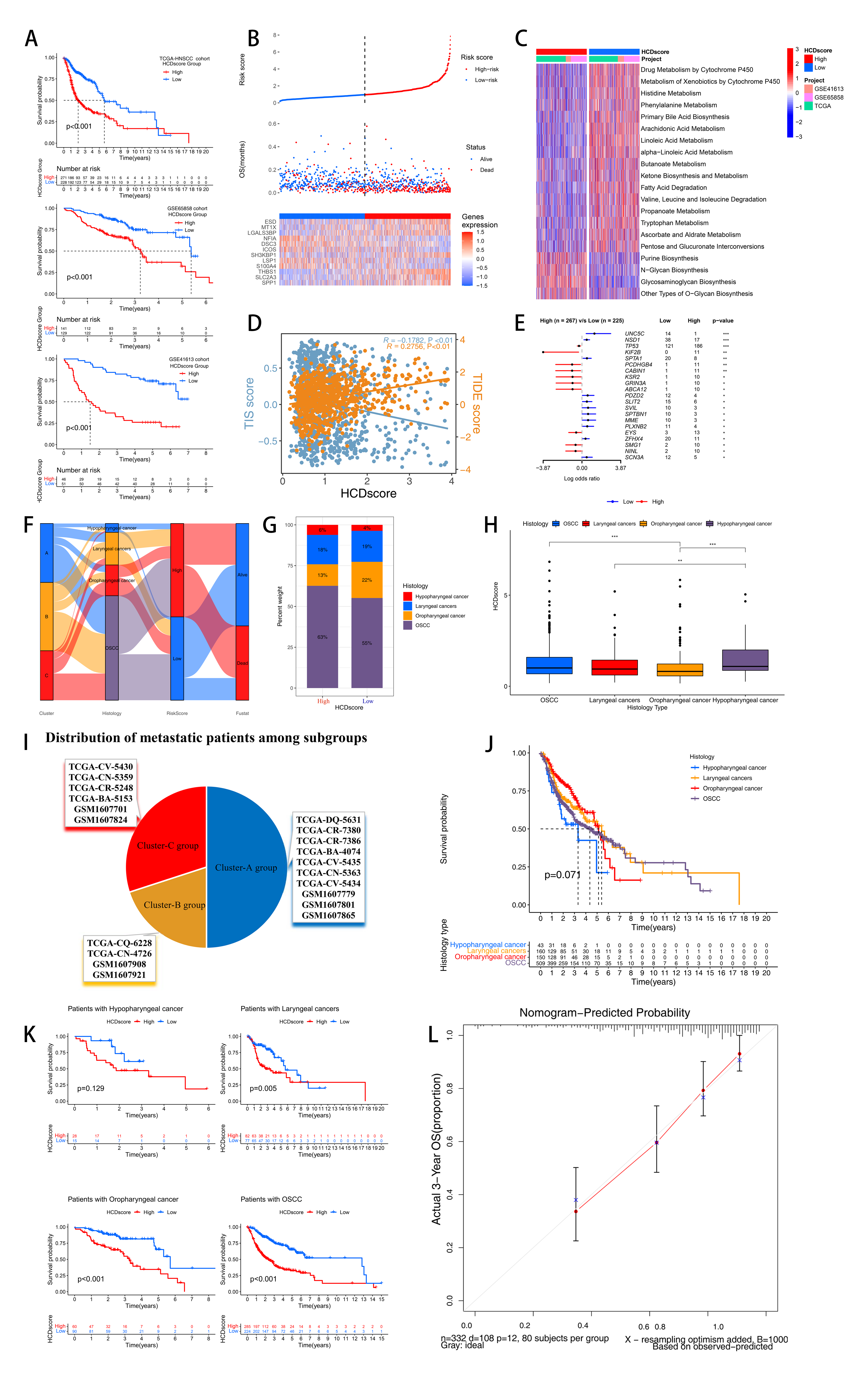

Supplement: Supplementary Figure S5 — Histological type analysis and verification of the accuracy of the nomogram. (A) Survival analyses for the low- and high-HCDscore groups in TCGA-HNSC, GSE65858 and GSE41613 cohorts, respectively. The optimal cutpoint for the levels of HCDscore was determined by R package “survminer”. (B) Distribution of HCDscore, different clusters of survival status and survival time between the high- and low-HCDscore groups. Heatmap shows the expression levels of the 12 hub genes for HNSCC patients. (C) GSVA enrichment analysis shows the activation states of metabolism-related biological pathways in high- and low-HCDscore groups. (D) The correlation between TIDEscore or TISscore and HCDscore by Spearman correlation analysis. (E) Forest plot of the differentially mutated genes between high- and low-HCDscore groups. (F) Alluvial diagram shows the changes of cluster, histology type, HCDscore level and survival outcomes. (G) The proportion of HNSCC patients with different clinical histopathological type. (H) Boxplot shows differences in HCDscore among different clinical histopathological type in HNSCC patients. The statistical difference of clusters was compared using the Kruskal–Wallis test. (I) Pie chart of the HNSCC patients with metastasis distribution among three clusters. (J) Survival analyses for HNSCC patients with each clinical histopathological phenotype using Kaplan–Meier curve and Log-rank test. (K) Survival analyses for each histology type including in HNSCC patients using Kaplan–Meier curve and Log-rank test. (L) The calibration plot for internal validation of the nomogram. [file Image_5.tif]
